# Socio-Economic Burden of Myasthenia Gravis: A Cost-of-Illness Study in Bulgaria

**DOI:** 10.3389/fpubh.2022.822909

**Published:** 2022-03-03

**Authors:** Valentina Ignatova, Kostadin Kostadinov, Evguenia Vassileva, Naira Muradyan, Georgi Stefanov, Georgi Iskrov, Rumen Stefanov

**Affiliations:** ^1^Clinic of Neurology, National Cardiology Hospital, Sofia, Bulgaria; ^2^Department of Social Medicine and Public Health, Faculty of Public Health, Medical University of Plovdiv, Plovdiv, Bulgaria; ^3^Clinic of Neurology, University Hospital “Tsaritsa Yoanna–ISUL”, Sofia, Bulgaria; ^4^Department of Neurology, Faculty of Medicine, Medical University of Sofia, Sofia, Bulgaria; ^5^Institute for Rare Diseases, Plovdiv, Bulgaria

**Keywords:** myasthenia gravis, cost-of-illness, socio-economic burden, burden of disease, societal costs, Bulgaria

## Abstract

**Background:**

Myasthenia gravis (MG) is a chronic autoimmune disorder, which is characterized by fatigable muscle weakness with frequent ocular signs and/or generalized muscle fatigue, and occasionally associated with thymoma. MG patients and their families face a significant socio-economic burden. This population is often experiencing unemployment, unwilling job transfers and decreased income.

**Objective:**

This study aimed to estimate the annual costs from a societal perspective in a triple dimension of direct health care costs, direct non-health care costs (formal and informal care) and labor productivity losses in MG patients from Bulgaria, as well as to identify the main clinical and demographical cost drivers.

**Methods:**

A bottom-up, cross-sectional, cost-of-illness analysis of 54 adult MG patients was carried out in 2020. To collect data on demographic characteristics, health resource utilization, informal care and productivity losses, questionnaires were administered to and completed by patients.

**Results and Conclusion:**

Median annual costs of MG in Bulgaria were 4,047 EUR per patient. Direct costs slightly outweighed indirect costs, with drugs cost item having the biggest monetary impact. Despite the zero-inflated median, hospitalizations also influenced the direct costs by an estimated amount of 1,512 EUR in the 3rd quartile. Social services and professional caregiver costs were found to be almost missing, with the vast majority of patients reporting reliance on informal caregivers. Severe generalized disease, disease crises, and recurrent infections were confirmed as statistically significant cost driving factors. There were no severe generalized MG patients in the bottom quartile of the total costs distribution. It should be noted that in both cases of crises or infections, the overall increase in the total costs was mainly due to higher indirect costs observed. Reliance on family members as informal caregivers is routine among Bulgarian MG patients. This phenomenon is likely due to the lack of access to appropriate social services. Moreover, it is directly related with higher disease burden and significant inequalities. There is a need for further research on MG in Bulgaria in order to design targeted health policies that meet the needs and expectations of these patients.

## Introduction

Myasthenia gravis (MG) is an autoimmune disorder, which is characterized by fatigable muscle weakness with frequent ocular signs and/or generalized muscle fatigue, and occasionally associated with thymoma (1). MG is caused by the presence of antibodies against components of the muscle membrane on the neuromuscular junction. Autoantibodies against the acetylcholine receptor (AChR) are the most common ones (2). However, antibodies against muscle-specific kinase (MuSK) and lipoprotein receptor-related protein 4 (LRP4) could be also found (3). The Medical Scientific Advisory Board (MSAB) of Myasthenia Gravis Foundation of America (MGFA) classifies MG into several categories: ocular (mild/moderate/severe), generalized, and associated with other diseases (4). The rationale of this classification is to identify patient subgroups, who share distinct clinical features or severity of disease that may indicate different prognosis or treatment response. Other approaches group specific subtypes of autoimmune MG subtypes according to the antigen target.

Prevalence of MG is estimated to be 1/5,000 in Europe, with incidence ranging between 1/250,000 and 1/33,000 (5). Incidence rates have a bimodal distribution in women, with peaks around the age of 30 and 50 years, respectively. In men, incidence increases steadily with age, with the highest rates observed in the range of 60 to 90 years of age (6). Infections and certain medications are considered factors for acute MG exacerbations requiring urgent medical attention and warrant caution (7). Other autoimmune diseases could contribute to MG relapse. Age of disease onset is a significant predictor of ocular MG generalization. Thymus hyperplasia and ptosis or diplopia could predict early generalization of ocular MG (8).

MG is a chronic condition, with the majority of patients needing long-term and often life-long therapy (9). Clinical symptomatology and ongoing treatment could cause significant quality of life decrement. MG patients often suffer from limitations that could be related to disease severity, different kinds of muscle involvement and poor treatment effectiveness. The support that an individual could receive from other persons can have a mediating effect in enhancing his/her ability to perform daily activities (10). MG individuals and their families face a significant socio-economic burden. This population is often experiencing unemployment, unwilling job transfers and decreased income (11).

Socio-economic burden is nowadays an important decision-making criterion in public health deliberations. Cost-of-illness studies are used to quantify the socio-economic impact of illnesses on both individuals and society as a whole. Socio-economic burden is a critical issue, as it reflects both direct and indirect costs to the health system, including the broader spillover effect on patients' families and caregivers. Health authorities and payers might benefit from measuring and comparing socio-economic impact of different disorders in order to optimize priority-setting and resource allocation. So long, the focus of assessment and evaluation has been mostly on cost-effectiveness and budget impact, with all other factors being pushed to the side. In this context, burden-of-disease data and explicit consideration of socio-economic burden could be very useful in policy-making and coverage decisions in particular (12).

This study aimed to estimate the annual costs from a societal perspective in a triple dimension of direct health care costs, direct non-health care costs (formal and informal care) and labor productivity losses in MG patients from Bulgaria, as well as to identify the main clinical and demographical cost drivers.

## Materials and Methods

### Study Design and Subjects

This is a cross-sectional study of adults (aged 18 years or more), who were diagnosed with MG, received outpatient care, and were living in the community. The fieldwork was carried out between May 2020 and September 2020. MG patients were invited to participate by their treating physician. All participants were informed about the study objectives and were asked to indicate their agreement to participate. Questionnaires were anonymous, with no personal data being collected. Information source used in the study was the self-completed questionnaire filled out by patients. According to national guidelines, ethics committee approval was not necessary for this type of study.

### Costing Methodology

Costing methodology of this study and overall design of the data collection process were based on the “Social Economic Burden and Health-Related Quality of Life in Patients with Rare Diseases in Europe” (BURQOL-RD) Project (13). Prevalence approach was used to estimate MG costs from a societal perspective. Disease prevalence considers all existing cases during a given year and all health care resources used for prevention, treatment and rehabilitation, as well as other resources used (formal and informal care) or lost (labor productivity) within that year because of the illness considered. Prevalence-based cost-of-illness analysis has the advantage of incorporating measurements of total annual health care expenditure, which is particularly relevant for chronic conditions such as MG that require long-term treatment. In this context, a bottom-up costing approach was applied to estimate total and average annual costs (14).

Data on resource utilization were collected for each patient. Unit costs of services and products that are funded by public funds were obtained from public health care cost databases. Unit costs of services and products that are not funded by public funds were based on average market prices. Unit costs were then multiplied by the resource quantities to estimate the annual cost per patient with 2020 as the reference year (15).

Informal care is defined as a group of tasks or care provided by non-professional caregivers (most often relatives), who are not paid for the provided care. Information about informal care was obtained from the questionnaire items concerning the time spent helping the patient with his/her basic daily living activities. The approach used to quantify the care hours was the proxy good method, which values time as an output. This method estimates the care provided by the informal caregiver considering that if he/she did not provide these services, their presence would have to be substituted by another person who could provide them (16).

Data on loss of labor productivity were obtained from physical units converted into monetary units through a human capital-based approach. Study estimates were based on average earnings (gross wages) of a worker for the year 2020.

### Statistical Analysis

Descriptive statistics and percentage distributions were applied. Median and interquartile range were presented, as study variables follow skewed distribution. Ordinary least squares linear regression (OLS) was used to identify clinical and demographic variables influencing total costs. A set of univariates, as well as multivariate regression models, including age and gender as covariates, were fit to the data. To normalize the outcome variable natural log transformation was applied. As log-level regression was used and no predictor variables were transformed, the coefficients were exponentiated of the independent variables with a base of *e*. Thereupon the coefficients were interpreted as percent change. Additionally, patients were assigned to ordinal categories according to the quartiles of total costs distribution into which they fell. This approach allows calculating the odds ratio (OR) of falling into the 3rd quartile over falling into the 1st one, by applying multinomial logistic regression using age and gender as covariates. Regression tree analysis was used to determine the importance of various social and clinical features in their ability to make the greatest difference in total costs. Cross validation tree pruning was used to determine tree depth. Analyses were performed using Microsoft Excel and R v.4.1 software packages.

## Results

### Socio-Demographic Profile of the Respondents

Fifty-four adult MG patients fully completed the study's questionnaire. Participants' mean age was 45 years (Standard deviation, SD = 13; Table 1). Female respondents represented about 80% of the sample. While the overall level of educational attainment was high, only 44% of the patients reported partial or full employment. More than a third of the participants (37%) were either unemployed or retired due to permanent disability.

**Table 1:** Socio-demographic profile of the respondents.

**Variable**	***n* (%)**
Age in years, mean (SD)	45 (13)
Gender, female	44 (81)
Marital status	
Single	9 (17)
Married	26 (48)
Cohabitation	9 (17)
Separated	1 (2)
Divorced	4 (7)
Widow	5 (9)
Educational attainment	
Primary	3 (6)
Secondary	31 (57)
Higher	20 (37)
Employment status	
Employed	24 (44)
Student	3 (6)
Unemployed	7 (13)
Retired	7 (13)
Permanent disability retired	13 (24)

### Clinical Profile of the Respondents

Mean disease duration was 11 year (SD = 10), with a mean age at diagnosis of 34 years (SD = 16; Table 2). The most prevalent type of MG was moderate generalized disease, with comorbidities reported by about half of the patients. Double vision was found in 74% of the sample and was the main MG symptom in 22% of the cases. The majority (61%) reported MG crises, with 56% of the patients experiencing such events at least twice a year.

**Table 2:** Clinical profile of the respondents.

**Variable**	***n* (%)**
Age in years at symptoms onset, mean (SD)	33 (16)
Age in years at diagnosis, mean (SD)	34 (16)
Diagnostic delay in months, mean (SD)	16 (32)
Age in years at thymectomy, mean (SD) (*n* = 24)	26 (12)
Disease duration in years, mean (SD)	11 (10)
MG Type	
Ocular	10 (19)
Generalized mild	13 (24)
Generalized moderate	22 (41)
Generalized severe	7 (13)
MG associated with other diseases	2 (4)
Main symptom	
Double vision	12 (22)
Swallow problems	16 (30)
Muscle weakness	24 (44)
Ptosis of eyelid	2 (4)
Type of disease crisis	
Myasthenic crisis	23 (43)
Cholinergic crisis	1 (2)
Mixed crisis	9 (17)
No crises	21 (39)
Frequency of disease crises	
No crises	24 (44)
Every 6 months	19 (35)
Every 3 months	7 (13)
Monthly	3 (6)
Weekly	1 (2)
Recurrent infections, yes	25 (46)
Comorbidities, yes	29 (54)
Double vision, yes	40 (74)
Thymectomy, yes	24 (44)

There was a significant association between MG type and main symptom (*p* = 0.008). Double vision was predominant in 60% of the patients with ocular MG, whereas muscle weakness was most common in patients with mild (77%) or severe (57%) generalized disease. A strong association was also found between MG type and crisis frequency (*p* < 0.001). Patients with severe (OR = 23.55; 95% CI 2.5–55; *p* = 0.013) or moderate generalized disease (OR = 21.81; 95% CI 3.4–44; *p* = 0.013) were more likely to experience crises than those with ocular MG. A similar pattern was found regarding the association between MG type and thymectomy (*p* = 0.019). Patients diagnosed with severe (OR = 22.5; 95% CI 1.6–31; *p* = 0.036) or moderate generalized disease (OR = 10.8; 95% CI 1.6–22; *p* = 0.036) were more likely to be thyroidectomized compared to those with ocular MG.

### Socio-Economic Burden of Myasthenia Gravis

Median annual costs of MG in Bulgaria were 4,047 EUR per patient (Table 3). Direct costs slightly outweighed indirect costs, with drugs cost item having the biggest monetary impact. Despite the zero-inflated median, hospitalizations also influenced the direct costs by an estimated amount of 1,512 EUR in the 3rd quartile. Visits costs came third. This item accounted for median annual costs of 194 EUR. Social services and professional caregiver costs were found to be almost missing. Although a substantial number of MG patients indicated a need for a caregiver and/or having a disability certificate, only one respondent reported use of professional (paid) caregiver. Surveyed individuals relied much more on informal caregivers (*p* = 0.019).

**Table 3:** Socio-economic burden of MG in Bulgarian adult patients.

**Annual costs (EUR, 2020)**	**Total number of respondents** ***n*** **=** **54**		
	**Median** **(interquartile range)**	**Range**	**No costs reported, *n* (%)**
Direct costs	1,366 (792–5,275)	0–12,843	1 (2)
Drugs	771 (443–780)	0–771	4 (7)
Tests	28 (0–77)	0–956	15 (28)
Visits	194 (95–425)	0–3,744	10 (19)
Hospitalizations	0 (0–1,512)	0–3,764	32 (59)
Materials	0 (0–0)	0–215	52 (96)
Transport	20 (0–100)	0–1,500	15 (28)
Social services	0 (0–0)	0–200	51 (94)
Professional caregiver	0 (0–0)	0–892	53 (98)
Informal caregiver	0 (0–0)	0–4,005	46 (85)
Indirect costs	0 (0–5,665)	0–8,193	30 (56)
Productivity loss	0 (0–0)	0–8,193	43 (80)
Early retirement	0 (0–0)	0–5,665	41 (76)
Total costs	4,047 (862–9,544)	443–17,884	–

Linear regression analysis found employment status as the only socio-demographic factor to significantly impact the total amount of costs in MG (Table 4). Employed respondents had 42.3% lower costs than other patient subgroups (β = −1.39; 95% CI −2.01 to −0.78; *p* < 0.01). Among the clinical factors considered, severe generalized disease, disease crises and recurrent infections were confirmed as statistically significant cost driving factors. There were no severe generalized MG patients in the bottom quartile of the total costs distribution. Respondents reporting crises regardless of their age and gender were nearly 8 times more likely to fall into the top quartile of the total costs distribution, compared to those with no crises (OR = 7.96; 95% CI 1.37–46; *p* = 0.02).

**Table 4:** Total costs in MG–OLS regression and quartiles logistic regression.

**Variable**	**β coefficient**	**% Difference**	**95% CI**	***P***	**OR (Q3/Q1)**	**95% CI**	***P***
Age	0.01	1.5	−0.01; 0.04	0.30	1.03	0.97; 1.09	0.36
Gender (female vs. male)	−0.35	−29.5	−1.23; 0.35	0.43	0.61	0.09; 4.37	0.62
Married and cohabitation vs. other^*^	−0.11	−10.4	−0.72; 0.70	0.90	1.32	0.28; 6.15	0.72
Education (university vs. lower)^*^	−0.55	−42.3	−1.31; 0.17	0.13	0.34	0.06; 1.71	0.19
Employment (employed vs. none)^*^	−1.39	−75.1	−2.01; −0.78	<0.01	0.07	0.01; 0.49	0.01
Comorbidities (yes vs. no)^*^	0.42	52.2	−0.32; 1.16	0.26	1.36	0.27; 6.84	0.70
Generalized severe form vs others^*^	1.11	203.4	0.12; 2.10	0.03	NA		
Muscle weakness vs. others^*^	0.42	52.2	−0.29; 1.13	0.24	2.28	0.46; 11.34	0.31
Double vision (yes vs. no)^*^	0.53	69.9	−0.28; 1.34	0.19	6.63	0.63; 70.12	0.12
Crises (yes vs. no)^*^	0.81	124.8	1.36; 1.49	0.02	7.96	1.37; 46	0.02
Infections (yes vs. no)^*^	0.80	122.5	0.11; 1.48	0.02	6.82	1.16; 39.9	0.03
Thymectomy (yes vs. no)^*^	0.41	50.7	−0.32; 1.13	0.27	3.86	0.69; 1.37	0.12

* *Age and gender adjusted coefficients*.

**Figure 1:**
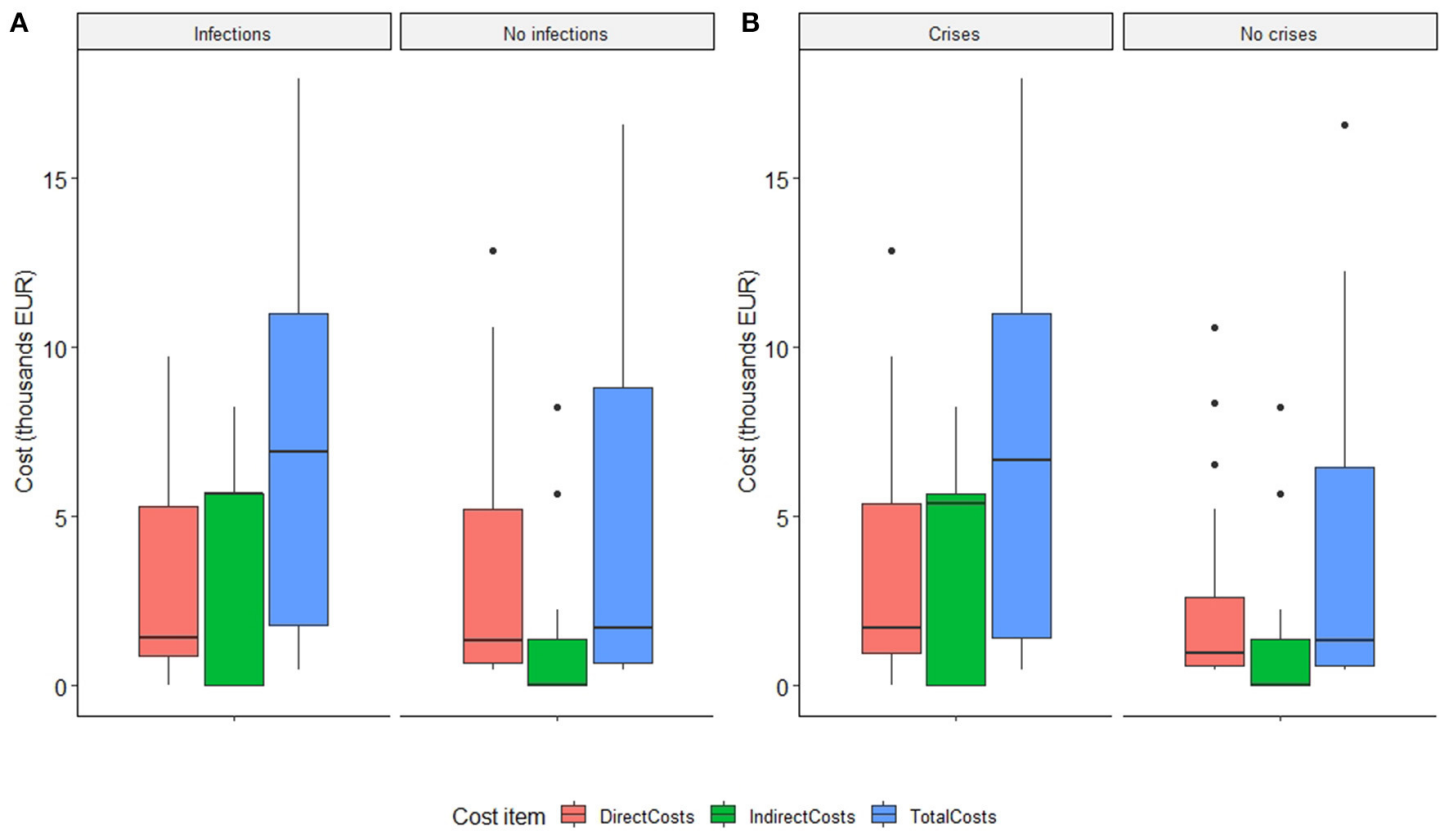
Distribution of socio-economic burden in MG by infection rate **(A)** and crisis rate **(B)**.

Results suggest an ordinal trend in the distribution of total costs relative to the reported frequency of crises. Estimated costs followed an ascending order as the frequency of crises increases (*p* = 0.007). After adjusting for gender and age, recurrent infections were also confirmed as a significant cost driver, with a similar ordinal trend (*p* = 0.028). Total costs in patients reporting infections were almost 123% higher than those in patients without infections. It should be noted that in both cases of crises or infections, the overall increase in the total costs was mainly due to higher indirect costs observed (Figure 1).

Despite the lack of statistical significance, higher costs were also found in individuals with lower educational attainment, as well as in those patients living alone. Comorbidities seem to contribute to higher costs too. All these factors may indeed generate significant health and social inequalities across the MG patient population.

**Figure 2:**
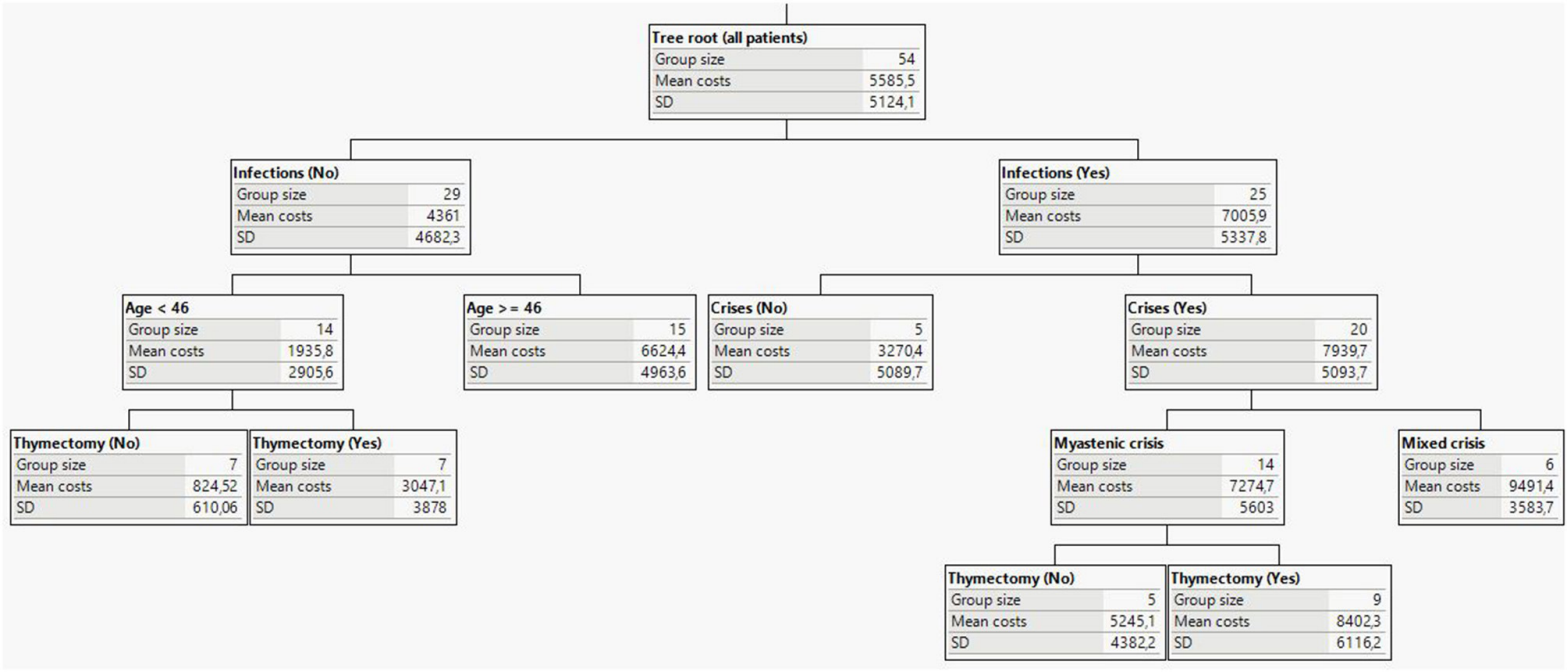
Decision tree analysis and main predicting factors for MG socio-economic burden (costs in EUR).

Regression tree analysis confirmed recurrent infections as the most important determinant of total cost differences in MG (Figure 2). This predictor was most able to discriminate total costs in the patient cohort. In the group of patients without infections, the greatest difference in costs was attributed to age. In patients younger than 46 years, the next splitting factor was thymectomy. Patients at this node and history of thymectomy had a mean cost of around 3,047 EUR in contrast to patients without thymectomy history, who had the lowest mean cost of 825 EUR. It is worth noting that the observed difference could be due to the more serious clinical presentation in patients with a history of thymus removal. Within the subgroup of patients reporting infections, the next important cost driver was disease crisis. The highest cost values were observed in the node of patients with myasthenic crises, infections and thymectomy.

## Discussion

MG is a chronic progressive neurological disorder. Life expectancy and quality of life highly depend on the timely access to and affordability of specialized medical care. The overall socio-economic burden of this disease is very much related to the organization and functioning of the health system (17). In this context, the Bulgarian health service is a highly centralized insurance system. The single health insurance fund (National Health Insurance Fund, NHIF) covers medical and dental services included in the benefit package, as well as medications listed in the Positive Drug List (PDL) (18). Three of the long-term medicinal therapies for MG management–prednisolone, azotitprine, and cyclosporine are currently not reimbursed for this indication by NHIF. Rituximab, an approved immunotherapy for stimulating long-term remission in MG, is not covered either. This has important implications for both patients' outcomes and MG's overall socio-economic burden. While the first three treatment options are usually paid out-of-pocket by the patients themselves, rituximab is virtually inaccessible due to its high market price.

Lack of timely access to appropriate and effective treatment could lead to poorer health outcomes and overall deterioration in the MG patients' condition. Our analysis found hospitalizations as one of the most important cost driving factors in MG. Although the median value for this cost item was 0 EUR, the overall spread was significant. It indicates a dramatic increase of the socio-economic burden in those MG patients, who require frequent hospitalizations. Costs for treatment of myasthenic crises were substantially higher in the case of intravenous immunovenin administration. Previous studies have confirmed similar results, with post-thymectomy crises and related hospitalizations being the largest cost driver (19, 20). Myasthenic crises and infections lead to more frequent and longer hospitalizations, thus impacting the overall socio-economic burden of MG (21).

A similar finding was observed regarding the amount of indirect costs. Again, the median value was 0 EUR. Despite that, indirect costs accounted for 46% of the overall MG socio-economic burden in Bulgaria. This was mainly a result from the most severe cases of MG from our study's sample. In these individuals, the category of indirect costs went up to 5,665 EUR, thus exceeding the direct costs. This insight should be discussed together with the restricted access to social services and professional caregivers. Only 3 and 1 respondents, respectively reported using such services. This is unlikely to be due to a lack of medical need for such services. Instead, this outcome is probably because of the lack of appropriate access scheme and the subsequent inability to afford.

This observation is indirectly confirmed by the share of MG patients who indicated use of informal caregiver. We found this indicator higher than the ones for the use of social services and formal caregivers. Given that informal care accounts for the majority of the day-to-day management of chronic disease patients, especially in the case of children and elderly individuals, further research on the magnitude of this cost category in MG is warranted. Future studies should focus on productivity loss and early retirement (both in patients and caregivers), as well as on informal care costs as separate components of the overall burden of disease. This is important, as there is a potential risk for spillover of burden on informal caregivers and family members (22).

This study's results though are subject to several limitations. First, the sample size was relatively small. According to a recent review of reimbursement claim data, the number of MG patients in Bulgaria was 1,283 in 2019 (23). However, it must be taken into account that our research was conducted during the COVID-19 pandemic, with very restricted access to potential respondents. An important advantage of our study is the fact MG patients were invited to participate in this survey by their treating physician, hence increasing the validity and integrity of the data collection process.

Second, our study sample included only MG adult patients. Therefore, the estimated socio-economic burden reflects solely the direct and indirect costs incurred by this patient cohort in Bulgaria. Nevertheless, MG's impact and spillover effect could be much larger in reality. The original research framework used in the BURQOL-RD Project included also patients' primary caregivers (13). We decided not to include this group of respondents mainly due to the COVID-19 pandemic and the related restrictions. Additional argument here is the fact that the study's target group focused on adult patients only. MG in children is substantially different in terms of clinical course and prognosis, thus leading to different levels of disease burden.

Third, invitation to participate in this survey and data collection was done *via* treating physicians. Consequently, our study's sample included only patients whose therapy is fully or partially covered by NHIF and/or who are regularly followed up by a medical professional. This could potentially mean underrepresentation of mild MG cases who do not seek treatment. Subsequently, it could lead to overestimation of this disease' socio-economic burden. In spite of that, 39% of study's participants indicated no myasthenic crises. Since, to the best of our knowledge, there are no available epidemiological studies from Bulgaria on MG, it is difficult to assess the distribution of MG patient population by disease severity.

Finally, direct cross-country comparison of MG's socio-economic burden is not always appropriate because of differences in patient populations, level of prices and inflation. A recent systematic review found a wide range of MG expenditure estimates (17). Annual direct costs per patient were between 760 and 28,780 USD, with cost per hospitalization varying between 2,550 and 164,730 USD. Nevertheless, cost-of-illness studies could effectively identify spending trends and cost driving factors. These could inform local stakeholders and improve policy making. At international level, this research helps recognize and transfer best practices from one jurisdiction to another.

## Conclusion

This is the first study to estimate the socio-economic burden in MG patients from Bulgaria. We found direct and indirect costs equally contributing to the overall disease burden. However, the latter category appeared to be the key to understanding and managing the socio-economic impact of MG. Analyzing a number of socio-demographic and clinical factors, the overall increase in MG's total costs was almost always a result of the higher indirect costs observed. Restrictions and lack of access to effective treatments lead to poorer health outcomes and general deterioration in the condition of MG patients. This subsequently translates into higher need for a caregiver and potential loss of productivity.

Reliance on family members as informal caregivers is routine among Bulgarian MG patients. This phenomenon is likely due to the lack of access to appropriate social services. Moreover, it is directly related with higher disease burden and significant inequalities. There is a need for further research on MG in Bulgaria in order to design targeted health policies that meet the needs and expectations of these patients. This research of ours could serve as a model for other chronically debilitating diseases, whose patients often face common public health and socio-economic challenges.

## Data Availability Statement

The datasets presented in this article are not readily available because the raw data supporting the conclusions of this article are available upon request. Requests to access the datasets should be directed to Georgi Iskrov, iskrov@raredis.org.

## Ethics Statement

Ethical review and approval was not required for the study on human participants in accordance with the local legislation and institutional requirements. The patients/participants provided their written informed consent to participate in this study.

## Author Contributions

VI contributed to the conception and design of the study, recruitment of survey respondents, analyzing the data, and critical review of the manuscript. KK, GS, and GI contributed to the conception and design of the study, analyzing the data, and writing the draft manuscript. EV and NM contributed to the conception and design of the study, recruitment of survey respondents, and critical review of the manuscript. RS contributed to the conception and design of the study, analyzing the data, and critical review of the manuscript. All authors contributed to the article and approved the submitted version.

## Conflict of Interest

The authors declare that the research was conducted in the absence of any commercial or financial relationships that could be construed as a potential conflict of interest.

## Publisher's Note

All claims expressed in this article are solely those of the authors and do not necessarily represent those of their affiliated organizations, or those of the publisher, the editors and the reviewers. Any product that may be evaluated in this article, or claim that may be made by its manufacturer, is not guaranteed or endorsed by the publisher.
